# Nurse awareness of clinical research: a survey in a Japanese University Hospital

**DOI:** 10.1186/1471-2288-14-85

**Published:** 2014-07-02

**Authors:** Hiroaki Yanagawa, Shigemi Takai, Michiko Yoshimaru, Toshiko Miyamoto, Rumi Katashima, Kikue Kida

**Affiliations:** 1 Clinical Trial Center for Developmental Therapeutics, Tokushima University Hospital, Kuramoto-cho 2, Tokushima 770-8503, Japan; 2 Division of Nursing, Tokushima University Hospital, Kuramoto-cho 2, Tokushima 770-8503, Japan

**Keywords:** Nurses, Research ethics, Clinical research, Awareness, Contribution

## Abstract

**Background:**

Clinical research plays an important role in establishing new treatments and improving the quality of medical practice. Since the introduction of the concept of clinical research coordinators (CRC) in Japan, investigators and CRC work as a clinical research team that coordinates with other professionals in clinical trials leading to drug approval (registration trials). Although clinical nurses collaborate with clinical research teams, extended clinical research teams that include clinical nurses may contribute to the ethical and scientific pursuit of clinical research.

**Methods:**

As knowledge of clinical research is essential for establishing an extended clinical research team, we used questionnaires to survey the knowledge of clinical nurses at Tokushima University Hospital. Five-point and two-point scales were used. Questions as for various experiences were also included and the relationship between awareness and experiences were analyzed.

**Results:**

Among the 597 nurses at Tokushima University Hospital, 453 (75.9%) responded to the questionnaires. In Japan, registration trials are regulated by pharmaceutical affairs laws, whereas other types of investigator-initiated research (clinical research) are conducted based on ethical guidelines outlined by the ministries of Japan. Approximately 90% of respondents were aware of registration trials and clinical research, but less than 40% of the nurses were aware of their difference. In clinical research terminology, most respondents were aware of informed consent and related issues, but ≤50% were aware of other things, such as the Declaration of Helsinki, ethical guidelines, Good Clinical Practice, institutional review boards, and ethics committees. We found no specific tendency in the relationship between awareness and past experiences, such as nursing patients who were participating in registration trials and/or clinical research or taking a part in research involving patients as a nursing student or a nurse.

**Conclusions:**

These findings suggest that clinical nurses have only limited knowledge on clinical research and the importance to have chances to make nurses aware of clinical research-related issues is suggested to establish an extended research team. Because of the study limitations, further study is warranted to determine the role of clinical nurses in establishing a suitable infrastructure for ethical pursuit of clinical research.

## Background

Clinical research plays an important role in improving the quality of medical practice, including the approval of drugs and medical devices (designated “registration trials” in the present article). The Japanese infrastructure for registration trials has improved since the introduction of the Good Clinical Practice (GCP) standard in 1997 and plans for the promotion of registration trials by the Ministry of Health, Labor, and Welfare and the Ministry of Culture and Science of Japan. The contribution of clinical research coordinators (CRC) in registration trials is now widely recognized not only for practical pursuit, but also for quality assurance in these trials.

On the other hand, investigator-initiated health research is conducted based on Japanese governmental guidelines, such as the 2001 Ethical Guidelines for Human Genome and Gene Analysis Research, the 2002 Ethical Guidelines for Epidemiological Research, and the 2003 Ethical Guidelines for Clinical Studies. Pharmaceutical Affairs Law and GCP are not applied to other types of investigator-initiated clinical research (designated “clinical research” in the present article) in Japan. Ordinary, physicians play roles of investigators and the contribution of CRC to clinical research is still limited, mainly for financial reasons. To overcome this inferiority in infrastructure, increasing the contribution of health professionals other than physicians in clinical research and registration trials could be a suitable strategy, not only for practical reasons, but also in regards to ethical conduct. Among various health professionals, clinical nurses, such as ward-based clinical nurses, are major in number and are currently engaged in clinical practice primarily as a nursing team. Several studies have revealed nurses’ attitudes and knowledge regarding nursing research [1-3] and the possible contribution of nurses in clinical ethics [4,5]. However, little is known about nurses’ views and knowledge about clinical research and registration trials.

At Tokushima University Hospital, a rural teaching hospital in Japan, we have a “local” registration rule for investigators in registration trials and clinical research. Attendance at clinical trial seminars emphasizing research ethics, organized regularly by the Clinical Trial Center for Developmental Therapeutics (CTCDT), is mandatory for investigators. The registration rule for physicians was originally established in 2001 in response to the GCP as an original rule for maintaining the quality of trials at Tokushima University Hospital. In 2009, the Ethical Guidelines for Clinical Studies was revised and the coverage of the local rule expanded to all investigators in order to maintain the quality of clinical research at Tokushima University Hospital. Currently, the attendance of clinical nurses at clinical trial seminars is still limited.

We have already reported physicians’ view on registration trials [6], and considered understanding clinical nurses’ awareness of research, including “local” situations, may contribute to the establishment of a clinical research infrastructure. Therefore, we focused on clinical nurses and used questionnaires to survey their status at Tokushima University Hospital. Since past experiences may influence on nurses’ awareness, we included several questions concerning experiences related to registration trials, clinical research, and research involving patients.

## Methods

We assessed nurses’ awareness of health research, including registration trials and clinical research, in a cross-sectional study conducted at Tokushima University Hospital.

A questionnaire was designed for use in this study, and was first administered to six CRC of the CTCDT of Tokushima University Hospital and the questionnaire was revised according to their suggestions. The questionnaire was anonymous and contained six parts with 47 questions (see Additional file 1). The first part consisted of five demographic questions. The second part consisted of five questions to determine the nurse’s general awareness of registration trials, clinical research, and CRC. The third part consisted of 14 questions concerning registration trials (8 questions), clinical research (4 questions), and nursing research (2 questions). The fourth part consisted of 9 questions concerning research-related terminology. The fifth part included 4 questions concerning experiences related to registration trials, clinical research, and research involving patients. Two questions about view of nurses’ role and willingness to work as CRC were also included in the fifth part. The sixth part consisted of 8 questions related to the nurse’s experience with education. In questions to survey awareness in the second and the fourth part, a five-point scale (confident, quite aware, aware, less aware, and not aware), was used. In the third part, nurses were advised to check if they were aware of the each issue. In other questions, two-point scale (yes and no), was used. Some questions concerned “local” situations at Tokushima University Hospital.

The questionnaire was provided to matrons of outpatient clinics and wards of Tokushima University Hospital and delivered to clinical nurses on occasion such as clinic- or ward-based conferences and was collected anonymously in 2011.

Data were expressed as the mean ± SD or n (%). We compared the awareness of clinical research-related issues based on the nurses’ experiences, such as nursing patients who were participating in registration trials and/or clinical research, and taking a part in research involving patients as a nursing student or a nurse, and analyzed the differences using the χ^2^ test. P-values <0.05 were considered significant. All P-values were based on two-sided tests. All statistical analyses were carried out using SPSS software, version 21.0 (IBM SPSS Statistics Base Authorized).

This study was approved by the Ethics Committee of Tokushima University Hospital.

## Results

### Respondent characteristics

Among the 597 nurses at Tokushima University Hospital, 453 (75.9%) questionnaires were completed and included in this analysis. The respondents included 15 males (3.3%) and 432 females (95.4%); 6 (1.3%) respondents provided no answer for gender. Concerning area of work, 360 (79.5%) of the respondents worked in wards, 90 (19.9%) worked in outpatient clinics, and 3 (0.6%) provided no information. The age distribution of the respondents was as follows: 20–29 years (n = 186, 41.1%), 30–39 years (n = 122, 26.9%), 40–49 years (n = 74, 16.3%), ≥ 50 years (n = 57, 12.6%), no answer (n = 14, 3.1%). The total nursing experience was as follows: <1 year (n = 23, 5.1%), 1–4 years (n = 125, 27.5%), 5–9 years (n = 86, 19.0%), 10–14 years (n = 55, 12.1%), 15–19 years (n = 44, 9.7%), 20–24 years (n = 33, 7.3%), 24–29 years (n = 29, 6.4%), ≥30 years (n = 41, 9.1%), no answer (n = 17, 3.8%). The nursing experience at Tokushima University Hospital was as follows: <1 year (n = 53, 11.7%), 1–4 years (n = 174, 38.3%), 5–9 years (n = 86, 19.0%), 10–14 years (n = 36, 7.9%), 15–19 years (n = 24, 5.3%), 20–24 years (n = 13, 2.9%), 24–29 years (n = 26, 5.7%), ≥30 years (n = 33, 7.3%), no answer (n = 8, 1.8%). The median values for all respondents are provided with ranges in Table 1.

**Table 1:** Respondent age, nursing experience, and experience at Tokushima University Hospital

	**Median (range), years**
Age	32 (20–61)
Total nursing experience	9.0 (<1 – 41)
Nursing experience at Tokushima University Hospital	4.7 (<1 – 41)

### General awareness of registration trials, clinical research, and CRC

In Japan, registration trials are regulated by pharmaceutical affairs laws. In contrast, clinical research is conducted based on the ethical guidelines of the ministries of Japan.

Therefore, we asked nurses regarding their awareness of registration trials and clinical research and the differences between them. As shown in Table 2, most respondents were aware (confident, quite aware, or aware) of registration trials (95.1%) and clinical research (87.9%), but only 36.6% of the respondents were aware (confident, quite aware, or aware) of their difference. As for CRC, 55.6% of the respondents were aware (confident, quite aware, or aware) of CRC, but only 34.9% were aware of the role of CRC.

**Table 2:** General awareness of registration trials, clinical research, and CRC

	**Confident**	**Quite aware**	**Aware**	**Less aware**	**Not aware**	**No answer**
Registration trials	64 (14.1%)	264 (58.4%)	103 (22.7%)	21 (4.6%)	1 (0.2%)	0 (0.0%)
Clinical research	49 (10.8%)	229 (50.6%)	120 (26.5%)	50 (11.0%)	5 (1.1%)	0 (0.0%)
Difference between registration trials and clinical research	11 (2.4%)	60 (13.2%)	95 (21.0%)	220 (48.7%)	65 (14.3%)	2 (0.4%)
Presence of CRC	34 (7.5%)	136 (30.0%)	82 (18.1%)	124 (27.4%)	76 (16.8%)	1 (0.2%)
Role of CRC	18 (4.0%)	43 (9.5%)	97 (21.4%)	192 (42.4%)	101 (22.3%)	2 (0.4%)

### Awareness of issues related to registration trials, clinical research, and nursing research

As shown in Table 3, most respondents were aware of the following issues related to registration trials: “registration trials are necessary for drug registration”, “informed consent is essential for registration trials”, “refusal of registration trials causes no disadvantage”, and “participants can withdraw anytime”. On the other hand, ≤50% of respondents were aware that “review by institutional review board is mandatory”, “CRC support registration trials”, “some registration trials use placebo”, and “reward for participants is prepared in registration trials”.

**Table 3:** Awareness of issues related to registration trials, clinical research, and nursing research

	**Aware**	**Not aware**
1. Awareness of issues related to registration trials		
Registration trials are necessary for drug registration	401 (88.5%)	52 (11.5%)
Review by institutional review board is mandatory	194 (42.8%)	259 (57.2%)
CRC support registration trials	200 (44.2%)	253 (55.8%)
Informed consent is essential for a registration trial	422 (93.2%)	31 (6.8%)
Refusal of a registration trial causes no disadvantage	394 (87.0%)	59 (13.0%)
Some registration trials use placebo	232 (51.2%)	221 (48.8%)
Participants can withdraw anytime	345 (76.2%)	108 (23.8%)
Reward for participants is prepared in registration trials	221 (48.8%)	232 (51.2%)
2. Awareness of issues related to clinical research		
Clinical research includes research using labeled drugs	192 (42.4%)	261 (57.6%)
Review by ethics committee is mandatory	242 (53.4%)	211 (46.6%)
Governmental ethical guidelines are applied to clinical research	245 (54.1%)	208 (45.9%)
Institutional registration is mandatory for investigators at Tokushima University Hospital*	148 (32.7%)	305 (67.3%)
3. Awareness on issues related to nursing research		
Review by ethics committee is mandatory	370 (81.7%)	83 (18.3%)
Institutional registration is mandatory for nurse investigators at Tokushima University Hospital*	244 (53.9%)	209 (46.1%)

*Concerns the “local” rule at Tokushima University Hospital.

Concerning issues related to clinical research, ≤50% of respondents were aware of the four issues examined, and only 32.7% were aware of the “local” rule: “institutional registration is mandatory for investigators at Tokushima University”.

More than 80% of the respondents were aware that review by an ethics committee is mandatory for nursing research, and 53.9% of the respondents were aware that institutional registration is mandatory for nurse investigators at Tokushima University Hospital.

### Awareness of research-related terminology

**Figure 1:**
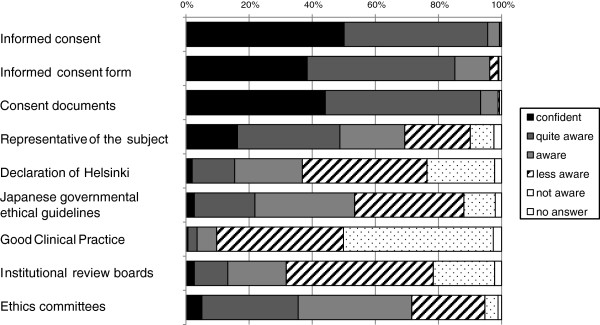
Awareness of research-related terminology.

Next, we asked nurses about their awareness of research-related terminology, including informed consent, informed consent form, consent documents, representative of the subject, Declaration of Helsinki, Japanese Governmental ethical guidelines, GCP, institutional review boards, and ethics committees (Figure 1). More than 95% of the respondents were aware (confident, quite aware, or aware) of informed consent and related issues (informed consent form and consent documents), 53.4% of the respondents were aware of Japanese governmental guidelines, and 9.7% of the respondents were aware of GCP. More respondents were aware of the ethics committees that review clinical research than of the institutional review boards that review registration trials (71.5% vs. 31.8%, respectively).

### Views and experience related to registration trials, clinical research, and research involving patients

As shown in Table 4, more than 90% of the respondents agreed that nurses need to know more about registration trials and clinical research, and 25.6% showed their willingness to work as CRC. We found that 30.2% of the respondents had experience nursing of patients who were participating in registration trials and/or clinical research. The respondents had experience taking a part in research involving patients as a nursing student (14.3%) or a nurse (36.9%).

**Table 4:** View and experience related to registration trials, clinical research, and nursing research

	**Yes**	**No**	**No answer**
It is necessary for nurses to know more about registration trials and clinical research	413 (91.2%)	35 (7.7%)	5 (1.1%)
I have experience nursing patients who were participating in registration trials and/or clinical research	137 (30.2%)	314 (69.4%)	2 (0.4%)
I have experience being asked by patients about registration trials and/or clinical research	59 (13.0%)	392 (86.6%)	2 (0.4%)
I am willing to work as CRC	116 (25.6%)	325 (71.8%)	12 (2.6%)
I have experience taking a part in research involving patients as a nursing student	65 (14.3%)	348 (76.8%)	40 (8.9%)
I have experience taking a part in research involving patients as a nurse	167 (36.9%)	246 (54.2%)	40 (8.9%)

### Influence of various experiences related to registration trials, clinical research, and research involving patients on awareness

Since awareness of nurses may vary depending on their past experience surveyed in the present study, we compared proportion of respondents that provided positive answer concerning awareness in subgroups. For this analysis, “confident”, “quite aware”, and “aware” were considered as positive answers concerning awareness. The subgroups were those with or without each experience, such as nursing patients who were participating in registration trials and/or clinical research, and taking a part in research involving patients as a nursing student or a nurse.

The results in categories of “general awareness of registration trials and clinical research” and “awareness of research-related terminology” are shown in Table 5. Although each subgroup with experience showed significantly higher proportion of respondents with positive answer in one or several issues in awareness than subgroup without experience, we could not find a certain tendency in influence of these experiences as a whole.

**Table 5:** Number and proportion of respondents that provided positive answer concerning awareness in subgroups (groups with or without various experiences)

	**Number (%) of respondents that provided positive answer**					
	**Experience nursing patients who were participating in registration trials and/or clinical research**		**Experience taking a part in research involving patients as a nursing student**		**Experience taking a part in research involving patients as a nurse**	
	**(+) (n = 137)**	**(−) (n = 314)**	**(+) (n = 65)**	**(−) (n = 348)**	**(+) (n = 167)**	**(−) (n = 246)**
1. General awareness						
Registration trials	137 (100%)	293 (93.3%)	59 (90.8%)	333 (95.7%)	164 (98.2%)*	228 (92.7%)
Clinical research	122 (89.1%)	275 (86.7%)	57 (87.7%)	307 (88.2%)	154 (92.2%)*	210 (85.4%)
Difference between registration trials and clinical research	66 (48.2%)**	99 (31.7%)	15 (23.1%)*	136 (39.1%)	76 (46.1%)**	75 (30.5%)
2. Awareness on research-related terminology						
Informed consent	136 (99.3%)	313(99.7%)	65 (100%)	348 (100%)	167 (100%)	246 (100%)
Informed consent form	133 (97.1%)	302 (96.2%)	61 (93.8%)	337 (97.4%)	160 (97.0%)	238 (96.7%)
Consent documents	135 (98.5%)	312 (99.4%)	64 (98.5%)	345 (99.7%)	167 (100%)	242 (99.2%)
Representative of the subject	110 (80.3%)**	204 (65.0%)	43 (66.2%)	243 (71.5%)	119 (73.5%)	167 (69.0%)
Declaration of Helsinki	57 (41.6%)	110 (35.0%)	24 (36.9%)	127 (37.4%)	66 (40.0%)	85 (35.4%)
Japanese governmental ethical guidelines	75 (54.7%)	167 (53.2%)	39 (60.0%)	183 (53.5%)	100 (60.6%)	122 (50.6%)
Good clinical practice	21 (15.9%)**	23 (7.3%)	9 (13.8%)	28 (8.3%)	17 (10.5%)	20 (8.3%)
Institutional review boards	54 (39.4%) *	90 (28.7%)	20 (30.8%)	111 (32.6%)	63 (38.4%)*	68 (28.2%)
Ethics committees	105 (76.6%)	219 (69.7%)	47 (72.3%)	252 (73.0%)	138 (83.1%)**	161 (66.0%)

*P < 0.05, **P < 0.01 compared to proportion for respondents without experience.

### Experience with health research education

We asked the nurses whether they had taken advantage of the various opportunities to learn about registration trials and clinical research at Tokushima University Hospital and their experience with nursing research. Although a considerable number of respondents had experience with nursing research education inside (38.9%) and outside (51.9%) Tokushima University Hospital, very few respondents (<10%) had experience with education regarding registration trials and clinical research (Table 6). We found that 21.6% of the respondents were aware of the clinical trial seminars organized regularly by the CTCDT of Tokushima University Hospital.

**Table 6:** Experience in health research education

	**Yes**	**No**	**No answer**
Experience participating in various seminars			
Orientation when starting work at Tokushima University Hospital	17 (3.8%)	411 (90.7%)	25 (5.5%)
Regular seminar organized by the Clinical Trial Center for Developmental Therapeutics	29 (6.4%)	399 (88.1%)	29 (6.4%)
Start-up meeting of registration trials at Tokushima University Hospital	20 (4.4%)	408 (90.1%)	25 (5.5%)
Seminar organized by the Clinical Trial Center for Developmental Therapeutics at wards	0 (0.0%)	428 (94.5%)	25 (5.5%)
Seminar on clinical research held outside of Tokushima University Hospital	7 (1.5%)	421 (92.9%)	25 (5.5%)
Seminar organized by the Nursing Department of Tokushima University Hospital	176 (38.9%)	272 (60.0%)	5 (1.1%)
Seminar on nursing research held outside of Tokushima University Hospital	235 (51.9%)	213 (47.0%)	5 (1.1%)
I know that the Clinical Trial Center for Developmental Therapeutics regularly hosts seminars on clinical research	98 (21.6%)	349 (77.0%)	6 (1.3%)

## Discussion

In a review concerning strategies for encouraging physician participation in clinical research, Rahman et al. [7] mentioned the importance of creating a research environment, such as a ‘centralized support services’ organization outside the physician group that facilitates the business of research by handling the clerical and other administrative tasks and communications among the research team. Although investigators participating in registration trials once performed all tasks related to the trial, from patient care to administrative work in Japan [8], a supporting division for clinical research including contribution of CRC is now widely accepted. For example, in a multicenter hypertension study, we found that physicians who recruit participants into a trial consider the presence of a support system with CRC as the reason to participate in the trial [9]. Therefore, the investigators and members of the supporting division of clinical research can be said to constitute a research team, at least in registration trials in Japan. To enable the full potential of the clinical research program, Baer et al. [10] encouraged collaboration among all individuals who support the program, including infusion nurses and pharmacists; because the direct benefit of clinical research may not be apparent to all medical professionals, efforts to promote support among staff are essential and awareness about clinical trials is needed on all levels of the institution and should be incorporated into the mission and vision of the site. In addition, a new plan for the promotion of clinical research and registration trials by the Ministry of Health, Labor, and Welfare and the Ministry of Culture and Science of Japan was introduced in 2012. Close communication between supporting divisions of clinical research, including CRC in Japan, and the nursing and pharmacy divisions allows a larger infrastructure to be established for clinical research and registration trials.

In the present study, ≥ 90% of the nurses were aware of registration trials and clinical research and agreed that nurses need to know more about registration trials and clinical research. The nurses were aware of the right of study participants to refuse and withdraw from registration trials, informed consent, and related issues (e.g., informed consent form and consent documents). These findings may reflect the familiarity of informed consent and related issues in clinical practice, and nurses may not be aware of the difference between these issues in clinical practice and in registration trials and clinical research. These possibilities should be examined in additional studies. Nevertheless, the nurses have considerable possibility of contributing to registration trials and clinical research in addition to centralized support services.

MacLean et al. [11] reported in a study on pediatric emergency nurses that the primary barriers to the nurses’ involvement in research is limited by research knowledge and experience, limited awareness and availability of research resources, lack of dedicated time, and limited recognition of research contributions. In agreement with this report, we found that nurses were not as aware of detailed issues in registration trials and clinical research, including the difference between registration trials and clinical research, some registration trial-related issues, clinical research-related issues, and some terminology. In the present study, we compared awareness in subgroups with or without respondents’ past experiences, such as nursing patients who were participating in registration trials and/or clinical research, and taking a part in research involving patients as a nursing student or a nurse, and no certain influence of these empirical experiences were observed. The findings may indicate that these empirical experiences had little impact to broaden nurses’ knowledge of clinical research-related issues. In contrast, systematic opportunities for education, such as seminars in and/or outside of the hospital, may contribute to nurses’ broader knowledge of clinical research. MacLean et al. [11] also reported that, in order to begin addressing the barriers, the Emergency Nurses Association developed a research curriculum based on the continuing education needs and interests identified by nurses in the United States. At Tokushima University Hospital, we have a local registration rule for clinical research investigators: attendance at clinical trial seminars, which includes basic issues affecting registration trials and clinical research at the postgraduate level, is mandatory if they ask the ethics committee to review their clinical research. The rule applies to nurses only when they work as investigators, such as when conducting their own research. Although 53.9% of the nurses in the present study were aware that institutional registration is mandatory for nurse investigators, their general awareness of the rule was low, and only a few had actually attended the institutional clinical trial seminar. Currently, voluntary attendance at the regular seminar is encouraged, but more opportunities for contact with the significance of clinical research should be considered.

In our previous survey at the First Symposium of the Shikoku Collaborative Group for Clinical Trials held in August 2009, a chance for learning, training, and person-to-person communication among personnel involved in clinical trials in the regional area, more support staff (including CRC) than medical staff exhibited willingness to contact staff from other medical institutions or organizations [12]. In a worldwide investigation of critical care research coordinators, “feelings of isolation” was mentioned among the “worst” aspects of their role [13]. In general, medical staff, such as ward-based clinical nurses, work as a nursing team, and CRC must be able to perform their research roles competently and must adapt to working alone as well as with a variety of clinical professionals. CRC often feel insecure, that they are perceived as a minority group, and that their complaints cannot be accepted by their colleagues who lack understanding and insight into the research process. In the present study, although the presence of CRC was recognized, clinical nurses were less aware of their role and the fact that CRC support registration trials. CRC communication with various professionals as an extended research team with clinical nurses could be a suitable strategy for lessening the feelings of isolation.

In a study of nursing staff involved in phase 1 oncology trials, Matsumoto et al. [14] reported that they encountered unique challenges because they were expected to be clinical trial specialists. Those who faced these challenges sometimes developed a negative attitude toward clinical trials, and the authors suggested the importance of education and support by other members of clinical trial teams to overcome the problem. Ethical issues arise for nurses involved in all phases of clinical trials, regardless of whether they are caregivers, research nurses, trial coordinators, or principal investigators [15]. These findings suggest that CRC communication with clinical nurses may have value for both groups.

Several limitations should be considered in the present study. First, the present study was conducted in one university hospital in Japan. Although almost Japanese hospitals has limited infrastructure for clinical research and clinical nurses are mainly engaged in clinical practice in Japan, the survey does not wholly reflect the awareness of nurses in Japan. Moreover, the health system and clinical research infrastructure vary among various countries, generalizability of the results in the present study in international settings should be examined in future studies. Second, the questionnaire was designed originally to survey the present awareness and experiences of nurses. Since awareness of nurses may vary depending on their past experience surveyed in the present study, we tried to compare awareness according to the respondents’ experiences. Although overall tendency was described in the present study, establishment of questionnaire that is suitable to compare these issues should be warranted.

## Conclusions

In spite of various limitations, we found that clinical nurses have only limited knowledge of clinical research, and experience nursing patients who participate in clinical research may result in a broader knowledge of clinical research. Nurses must have the opportunity to be aware of clinical research-related issues, and examining that nurses’ broader knowledge of clinical research can contribute to the development of extended research teams for clinical research. Because of the study limitations, further study is warranted to determine the role of clinical nurses in establishing suitable infrastructure for clinical research.

## Competing interests

The authors declare that they have no competing interests in relation to this article.

## Authors’ contributions

HY conceived of the study, collected data and analyzed them, and drafted the manuscript. ST and TM participated in the design of the study and collected data. MY contributed to the statistical analysis. RK and KK participated in the design of the study and helped to draft the manuscript. All authors read and approved the final manuscript.

## Pre-publication history

The pre-publication history for this paper can be accessed here:

http://www.biomedcentral.com/1471-2288/14/85/prepub

## Supplementary Material

Additional file 1 **Nurse awareness of clinical research questionnaire.** (English translation by the authors, originally written in Japanese). Click here for file

## References

[B1] Björkström ME Hamrin EK Swedish nurses’ attitudes towards research and development within nursing J Adv Nurs 2001 34 706 714 11380739 10.1046/j.1365-2648.2001.01800.x

[B2] Moreno-Casbas T Fuentelsaz-Gallego C Gil de Miguel A Gonzalez-Marıa E Clarke SP Spanish nurses’ attitudes towards research and perceived barriers and facilitators of research utilisation: a comparative survey of nurses with and without experience as principal investigators J Clin Nurs 2011 20 1936 1947 21539627 10.1111/j.1365-2702.2010.03656.x

[B3] Timmins F McCabe C McSherry R Research awareness: managerial challenges for nurses in the Republic of Ireland J Nurs Manage 2012 20 224 235 10.1111/j.1365-2834.2012.01333.x 22380417

[B4] Wocial LD Bledsoe P Helft PR Everett LQ Nurse ethicist: innovative resource for nurses J Prof Nurs 2010 26 287 292 20869028 10.1016/j.profnurs.2010.06.003

[B5] Cusveller B Nurses serving on clinical ethics committees: a qualitative exploration of a competency profile Nurs Ethics 2012 19 431 442 22323396 10.1177/0969733011426817

[B6] Yanagawa H Nishiya M Miyamoto T Shikishima M Imura M Nakanishi R Ariuchi N Akaishi A Takai S Abe S Kisyuku M Kageyama C Sato C Yamagami M Urakawa N Sone S Irahara M Clinical trials for drug approval: a pilot study of the view of doctors at Tokushima University Hospital J Med Invest 2006 53 292 296 16953067 10.2152/jmi.53.292

[B7] Rahman S Majumder MAA Shaban SF Rahman N Ahmed M Abdulrahman KB D’Souza UJA Physician participation in clinical research and trials: issues and approaches Adv Med Edu Pract 2011 2 85 93 10.2147/AMEP.S14103 PMC3661249 23745079

[B8] Ebihara A Takahashi K Ikemoto F Yamamoto K Clinical pharmacology and clinical trials in Japan J Mol Med 1996 74 479 486 8872862 10.1007/BF00217525

[B9] Yanagawa H Kishuku M Akaike M Azuma H Irahara M View of physicians on and barriers to patient enrollment in a multicenter clinical trial: experience in a Japanese rural area Int Arch Med 2010 3 7 20525325 10.1186/1755-7682-3-7 PMC2893519

[B10] Baer AR Zon R Devine S Lyss AP The clinical research team J Oncol Pract 2011 7 188 192 21886502 10.1200/JOP.2011.000276 PMC3092661

[B11] MacLean S Désy P Juarez A Perhats C Gacki-Smith J Research education needs of pediatric emergency nurses J Emerg Nurs 2006 32 17 22 16439282 10.1016/j.jen.2005.11.006

[B12] Yanagawa H Irahara M Houchi H Kakehi Y Moritoyo T Nomoto M Miyamura M Shuin T Shikoku Collaborative Group for Promotion of Clinical Trials View and present status of personnel involved in clinical trials: a survey of participants from the First Symposium of the Shikoku Collaborative Group for Promotion of Clinical Trials J Med Invest 2011 58 81 85 21372491 10.2152/jmi.58.81

[B13] Eastwood GM Roberts B Williams G Rickard CM A worldwide investigation of critical care research coordinators’ self-reported role and professional development priorities: the winner survey J Clin Nurs 2013 22 838 847 23039162 10.1111/j.1365-2702.2012.04230.x

[B14] Matsumoto K Nagamura F Ogami Y Yamashita N Kamibeppu K Difficulties of nursing staff involved in phase 1 oncology trials in Japan Cancer Nurs 2011 34 369 375 21242764 10.1097/NCC.0b013e31820809ad

[B15] Oberle K Allen M Ethical considerations for nurses in clinical trials Nurs Ethics 2006 13 180 186 16526151 10.1191/0969733006ne836oa

