# Supplementary material for: Nurse awareness of clinical research: a survey in a Japanese University Hospital
Source: BMC Med Res Methodol. 2014 Jul 2;14:85. doi: 10.1186/1471-2288-14-85 (PMC4091657; doi:10.1186/1471-2288-14-85)
Supplement: Additional file 1 — Nurse awareness of clinical research questionnaire. (English translation by the authors, originally written in Japanese). [file 1471-2288-14-85-S1.docx]

**Nurse awareness of clinical research questionnaire**

**(English translation by the authors, originally written in Japanese)**

Part 1

Concerning demographic data, please check or write in the followings.

1) Age

( ) years old,

2) Gender

□ male □ female

3) Area of work

□ ward □ outpatient clinic

4) Total nursing experience

( ) years

5) Nursing experience at Tokushima University Hospital

( ) years

Part 2

Are you aware of the followings? Please check one box.

|  | Confident | Quite aware | Aware | Less aware | Not aware |  |
| --- | --- | --- | --- | --- | --- | --- |
| 1) Registration trials |  |  |  |  |  |  |
| 2) Clinical research |  |  |  |  |  |  |
| 3) Difference between registration trials and clinical research |  |  |  |  |  |  |
| 4) Presence of CRC |  |  |  |  |  |  |
| 5) Role of CRC |  |  |  |  |  | 2 (0.4%) |

Part 3

1. Are you aware of the following issues related to registration trials?

Please check if you are aware of the each issue.

( ) Registration trials are necessary for drug registration.

( ) Review by institutional review board is mandatory.

( ) CRC support registration trials.

( ) Informed consent is essential for a registration trial.

( ) Refusal of a registration trial causes no disadvantage.

( ) Some registration trials use placebo.

( ) Participants can withdraw anytime.

( ) Reward for participants is prepared in registration trials.

2. Are you aware of the following issues related to clinical research?

Please check if you are aware of the each issue.

( ) Clinical research includes research using labeled drugs.

( ) Review by ethics committee is mandatory.

( ) Governmental ethical guidelines are applied to clinical research.

( ) Institutional registration is mandatory for investigators at Tokushima University

Hospital.

3. Are you aware of the following issues related to nursing research?

Please check if you are aware of the each issue.

( ) Review by ethics committee is mandatory.

( ) Institutional registration is mandatory for nurse investigators at Tokushima

University Hospital

Part 4

Are you aware of the followings? Please check one box.

|  | Confident | Quite aware | Aware | Less aware | Not aware |  |
| --- | --- | --- | --- | --- | --- | --- |
| 1) Informed consent |  |  |  |  |  |  |
| 2) Informed consent  form |  |  |  |  |  |  |
| 3) Consent documents |  |  |  |  |  |  |
| 4) Representative of  the subject |  |  |  |  |  |  |
| 5) Declaration of Helsinki |  |  |  |  |  | 2 (0.4%) |
| 6) Japanese governmental ethical guidelines |  |  |  |  |  |  |
| 7) Good Clinical Practice |  |  |  |  |  |  |
| 8) Institutional review boards |  |  |  |  |  |  |
| 9) Ethics committees |  |  |  |  |  |  |

Part 5

1. How do you think about the followings? Please check one box.

|  | Yes | No |
| --- | --- | --- |
| 1) It is necessary for nurses to know more about registration trials and clinical research. |  |  |
| 2) I have experience nursing patients who were participating in registration trials and/or clinical research. |  |  |
| 3) I have experience being asked by patients about registration trials and/ or clinical research. |  |  |
| 4) I am willing to work as CRC. |  |  |

2. Do you have following experience? Please check one box.

|  | Yes | No |
| --- | --- | --- |
| 1) I have experience taking a part in research involving patients as a nursing student |  |  |
| 2) I have experience taking a part in research involving patients as a nurse |  |  |

Part 6

1. Do you have following experience in health research education? Please check one box.

|  | Yes | No |
| --- | --- | --- |
| 1) Orientation when starting work at Tokushima University Hospital |  |  |
| 2) Regular seminar organized by the Clinical Trial Center for  Developmental Therapeutics |  |  |
| 3) Start-up meeting of registration trials at Tokushima University Hospital |  |  |
| 4) Seminar organized by the Clinical Trial Center for Developmental Therapeutics at wards. |  |  |
| 5) Seminar on clinical research held outside of Tokushima University Hospital |  |  |
| 6) Seminar organized by the Nursing Department of Tokushima University Hospital |  |  |
| 7) Seminar on nursing research held outside of Tokushima University Hospital |  |  |

2. Are you aware of the following fact? Please check one box.

|  | Yes | No |
| --- | --- | --- |
| 1) The Clinical Trial Center for Developmental Therapeutics regularly hosts seminars on clinical research. |  |  |
